# Synergistic effect between denosumab and immune checkpoint inhibitors (ICI)? A retrospective study of 268 patients with ICI and bone metastases

**DOI:** 10.1016/j.jbo.2024.100634

**Published:** 2024-09-21

**Authors:** E. Mabrut, S. Mainbourg, J. Peron, D. Maillet, S. Dalle, C. Fontaine Delaruelle, E. Grolleau, P. Clezardin, E. Bonnelye, C.B. Confavreux, E. Massy

**Affiliations:** a INSERM UMR 1033-LYOS, Lyon, France; b Université de Lyon, France; c Centre Expert des Métastases Osseuses (CEMOS) - Service de Rhumatologie, Centre Hospitalier Lyon-Sud, Hospices Civils de Lyon, Pierre-Bénite, France; d Service universitaire de Pharmacotoxicologie, Hospices Civils de Lyon, France; e Service d’Oncologie Médicale. Institut de Cancérologie des Hospices Civils de Lyon (IC-HCL), Hôpital Lyon Sud-HCL, Pierre-Bénite, France; f Service de Dermatologie, Hôpital Lyon Sud - HC L, Pierre-Bénite, France; g Service de Pneumologie, Hôpital Lyon Sud - HC L, Pierre-Bénite, France; h Université de Lille, France; i INSERM UMR9020-UMR127, Lille, France

**Keywords:** Bone metastasis, Immune check point inhibitors, Denosumab, Survival

## Abstract

**Background:**

Bone metastasis is a significant concern in advanced solid tumors, contributing to diminished patient survival and quality of life due to skeletal-related events (SREs). Denosumab (DMAB), a monoclonal antibody targeting the receptor activator of nuclear factor kappa-B ligand (RANKL), is used to prevent SREs in such cases. The RANK/RANKL axis, crucial in immunological processes, has garnered attention, especially with the expanding use of immune checkpoint inhibitors (ICI) in modern oncology.

**Objective:**

Our study aims to explore the potential synergistic antitumor effects of combining immunotherapy with denosumab, as suggested by anecdotal evidence, small cohort studies, and preclinical research.

**Methods:**

We conducted a retrospective analysis using the IMMUCARE database, encompassing patients receiving ICI treatment since 2014 and diagnosed with bone metastases. We examined overall survival (OS), progression-free survival (PFS) and switch of treatment line based on denosumab usage. Patients were stratified into groups: without denosumab, ICI followed by denosumab, and denosumab followed by ICI. Survival curves and multivariate Cox regression analyses were performed.

**Results:**

Among the 268 patients with bone metastases, 154 received treatment with ICI alone, while 114 received ICI in combination with denosumab at some point during their oncological history. No significant differences were observed in overall survival (OS) or progression-free survival (PFS) between patients receiving ICI monotherapy and those receiving ICI with denosumab (p = 0.29 and p = 0.79, respectively). However, upon analyzing patients who received denosumab following ICI initiation (17 patients), a notable difference emerged. The group receiving ICI followed by denosumab exhibited a significant advantage compared to those without denosumab (154 patients) or those receiving denosumab before ICI initiation (72 patients) (p = 0.022).

**Conclusion:**

This retrospective investigation supports the notion of potential benefits associated with sequential administration of ICI and denosumab, although statistical significance was not achieved. Future studies, including prospective trials or updated retrospective analyses, focusing on cancers treated with first-line immunotherapy, could provide further insights into this therapeutic approach.

## Highlights

• Bone metastasis significantly affects patient survival and quality of life in advanced solid tumors, and denosumab is used to prevent skeletal-related events in these cases.
• The study investigates the potential synergistic effects of combining immunotherapy with denosumab, leveraging anecdotal evidence and preliminary research.
• A retrospective analysis was conducted on patients with bone metastases receiving immune checkpoint inhibitors (ICI) from the IMMUCARE database.
• Results indicated no significant differences in overall survival or progression-free survival between ICI monotherapy and combined ICI-denosumab treatment.
• Notably, patients who received denosumab after starting ICI treatment showed a significant survival advantage, suggesting potential benefits from this sequential therapy approach.

## 1 Introduction

Cancer remains a global health challenge, with an estimated 18.1 million cases worldwide and 9.6 million new cases annually [1]. Metastasis to the bone emerges as a critical concern, ranking as the third most common site of metastasis and significantly worsening patient prognosis [2], [3], [4]. Bone metastasis introduce a range of complications known as skeletal-related events (SRE), including pathologic bone fractures, spinal cord compression, orthopedic surgical intervention, palliative radiation directed at the bone, and hypercalcemia. SRE profoundly affect patient quality of life [4], [5], [6], [7].

In order to prevent SRE, clinicians turn to antiresorptive treatments such as bisphosphonates and denosumab. Denosumab (DMAB), a fully monoclonal human IgG2 antibody, is specifically engineered to target the receptor activator of nuclear factor kappa-B ligand (RANKL).

The RANK/RANKL pathway, where RANK (Receptor Activator of Nuclear Factor Kappa-B) interacts with its ligand RANKL (Receptor Activator of Nuclear Factor Kappa-B Ligand), plays a pivotal role in bone metastasis, actively participating in the vicious circle of bone metastasis [8]. Notably, immune cells also express RANK and RANKL, suggesting broader implications of this pathway beyond neoplastic tissues [2], [9], [10], [11].

Conversely, Immune Checkpoint Inhibitors (ICI) have emerged as promising treatments over the past decade. ICI function by suppressing tumor-induced immune system inhibition mechanisms, thereby enhancing the immune system's ability to recognize and target cancer cells. This has been an important step forward in the management of many cancers.

Despite the promise of ICI, challenges such as resistance and variable efficacy persist across different cancer types. To address these challenges and improve treatment outcomes, novel therapeutic approaches are being explored.

Preclinical studies and case reports have hinted at a potential synergistic anti-tumor effect between ICI and DMAB [18], [12], [13], [14], [15], [16]. Combination therapy has been associated with increased production of Th1 cytokines and intra-tumoral CD8 + lymphocytes, indicating enhanced anti-tumor immune responses [12], [16], [17], [18]. Despite promising findings from preclinical models and case reports, the lack of well-designed prospective studies hampers the validation of these observations. Retrospective studies have shown promising but inconclusive results [14], [16], [19], [20], [21]. In light of this, we conducted a retrospective study involving a substantial cohort of 268 patients to evaluate the impact of DMAB on the oncological response to ICI therapy.

Our hypothesis posits that the sequential administration of ICI followed by DMAB may result in superior oncological outcomes compared to ICI monotherapy or the reverse sequence.

## 2 Methods

We conducted a monocentric retrospective observational study at the Lyon Sud University Hospital, Hospices Civils de Lyon, France. Eligible patients were adult patients with bone metastasis, receiving ICI for solid tumors or lymphoma from 2014 to 2019, and who were included in the IMMUCARE registry [22]. The study received the approval of the ethical review board of the Hospices Civils de Lyon (N° 22_452on June 13th, 2022) and Commission Nationale de l’Informatique et des Libertés (CNIL, French data protection authority, N° 19–021).

### 2.1 Data collection

Data from all patients treated with ICI therapy in the dermatology, oncology, or pneumology departments at the Hôpital Lyon Sud, Hospices Civils de Lyon, Pierre Bénite, France from January 2014 to December 2019 were retrospectively analysed. Data were extracted from medical charts using a standardized data collection form. Patient characteristics included age, sex, weight, height, Body Mass Index (BMI), smoking habits, alcohol use, bone metastatic status (single, oligometastatic or multimetastatic). Cancer characteristics encompassed tumor type, metastatic status at inclusion, diagnosis date, type of ICI used, date of ICI initiation and cessation, reason for ICI cessation, associated treatments, initial tumor response, date of progression, and date of death. Progression was evaluated by the patient’s treating oncologist. We also collected the use of denosumab, including the initiation date and duration of treatment, the use and duration of corticosteroids at the start of ICI therapy, and the use and duration of zoledronic acid, another anti-resorptive treatment. (Table 1).

**Table 1:** Patient characteristics according to the group of treatment (ICI alone, n = 154, ICI then DMAB, n = 17, DMAB then ICI, n = 72).

**Characteristics**	**ICI alone**, N=154^1^	**ICI then DMAB**, N=17^1^	**DMAB then ICI**, N=72^1^	**p-value**^2^
Age (years)	65.7 (10.5)	63.3 (11.2)	65.1 (9.3)	0.63
Men gender	108 (70 %)	13 (76 %)	50 (69 %)	0.84
Cancer type				0.060
Lung	121 (79 %)	15 (88 %)	64 (89 %)	
Melanoma	15 (9.7 %)	0 (0 %)	1 (1.4 %)	
Kidney	12 (7.8)	1 (5.9 %)	3 (4.2 %)	
Bladder	1 (0.6 %)	1 (5.9 %)	2 (2.8 %)	
Other	5 (3.2 %)	0 (0 %)	2 (2.8 %)	
Histologic status				0.045
Adenocarcinoma	86 (62 %)	10 (63 %)	53 (75 %)	
Epidermoid	33 (24 %)	2 (13 %)	9 (13 %)	
Clear cell tumor	10 (7.2 %)	0 (0 %)	2 (2.8 %)	
Poorly differenciated	3 (2.2 %)	2 (13 %)	3 (4.2 %)	
Neurendocrine	1 (0.7 %)	0 (0 %)	2 (2.8 %)	
Urothelial	0 (0 %)	1 (6.3 %)	2 (2.8 %)	
Large cell	1 (0.7 %)	0 (0 %)	0 (0 %)	
Agressive T lymphoma	1 (0.7 %)	0 (0 %)	0 (0 %)	
Unknown	15 (9.7 %)	1 (5.9 %)	1 (1.4 %)	
Treatment				0.006
Nivolumab	125 (81 %)	10 (59 %)	65 (90 %)	
Pembrolizumab	28 (18 %)	6 (35 %)	6 (8.3 %)	
Atezolizumab	0 (0 %)	1 (5.9 %)	1 (1.4 %)	
Pembrolizumab + nivolumab	1 (0.6 %)	0 (0 %)	0 (0 %)	
Number of metastatic sites	3.0 (1.6)	2.8 (1.5)	2.8 (1.2)	0.89
Metastatic site				
Lung	75 (49 %)	8 (47 %)	41 (57 %)	0.48
Liver	45 (29 %)	5 (29 %)	25 (35 %)	0.70
Bone	154 (100 %)	17 (100 %)	72 (100 %)	
Brain	47 (31 %)	4 (24 %)	16 (22 %)	0.45
Others	15 (9.7 %)	5 (29 %)	3 (4.2 %)	0.010
Zoledronic acid	20 (13 %)	3 (18 %)	3 (4.2 %)	0.063
Year of inclusion				
2018	69 (45 %)	9 (53 %)	16 (22 %)	
2017	49 (32 %)	5 (29 %)	27 (38 %)	
2016	26 (17 %)	0 (0 %)	18 (25 %)	
2015	9 (5.8 %)	3 (18 %)	11 (15 %)	
2014	1 (0.6 %)	0 (0 %)	0 (0 %)	

^1^Mean (SD)/ Median (IQR); n (%).

^2^Kruskal-Wallis rank sum test; Pearson's Chi-squared test; Fisher's exact test.

### 2.2 Population analyzed

We decided to first analyze patients with and without DMAB independent of sequence and temporal association (*total population*). Then, for patients with DMAB exposure, we kept those who had received combotherapy (ICI and DMAB) and excluded patients whose treatments did not overlap, as we wanted to study synergy. (*population combotherapy*).

Overlap was considered when prescription of DMAB and ICI were simultaneous.

**Fig. 1:**
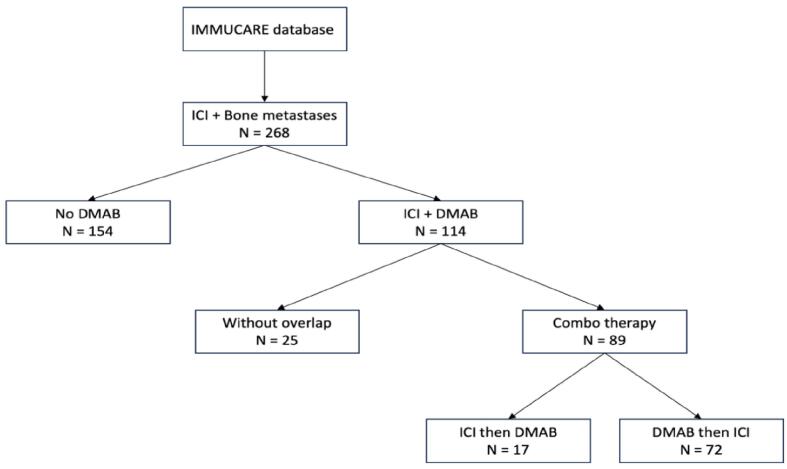
Flow chart: The flowchart begins with a cohort of 268 patients from the IMMUCARE database who have bone metastases and were treated with ICI. These patients are categorized into two main groups. The first group, comprising 154 patients, did not receive denosumab (DMAB), while the second group of 114 patients received both ICI and DMAB therapy. Fig. 2 present overall survival (OS) and progression-free survival (PFS) between these two groups. Within the group of patients who received both ICI and DMAB, they were further divided into two subgroups: 25 patients received the treatments sequentially with no overlap, meaning ICI and DMAB were administered at different times. The other subgroup of 89 patients received combination therapy for a significant period. Fig. 3 presents OS and PFS comparing the combotherapy group with ICI monotherapy group. Finally, the combotherapy group was split according to the sequence: 17 patients received ICI first, followed by DMAB, while 72 patients received DMAB first, followed by ICI. Fig. 4, present OS, PFS, and treatment line change between the sequence group.

We categorized patients based on the sequence in the combination therapy population: ICI followed by DMAB, or DMAB followed by ICI (*sequential population*). All of these patients had overlapping prescriptions of ICI and DMAB (Fig. 1).

All data collected from these 268 patients’ charts were collected by a single person who searched in rheumatology/oncology/stomatology records (EM).

### 2.3 Statistical analysis

We analyzed overall survival (OS), progression-free survival (PFS) and switch of treatment line in these different populations. Switch of treatment line corresponds to the moment when the patient stops his treatment. It therefore corresponds to the cessation of ICI use. Progression, poor tolerance and discontinuation of active therapies are all reasons for a switching line. It should be pointed that the suspension of treatment during therapeutic pauses is not considered as a change of line. Switch of treatment line is used as a substitute criterion and is interesting because it reflects both efficacy (progression, discontinuation of active therapies) and safety (adverse events, tolerance).

For safety analysis, we collected data on osteonecrosis of the jaws (ONJ) and occurrence of hypocalcemia after initiation of antiresorptive therapy.

The baseline characteristics of the patients were summarized using numbers and percentages for categorical variables and the median with range for continuous variables (Table 1).

Kruskal-Wallis test was utilized to compare multiple groups of continuous variables, while the Wilcoxon test was employed for comparing two groups of continuous variables. Fisher’s exact test was used for comparing categorical variables. Spearman's coefficient was utilized for correlation tests between variables. Survival analysis was conducted using the Kaplan-Meier method, and statistical comparisons were made using log-rank tests. All analyses were carried out using R software, version 4.2.1 (©2017; R Foundation for Statistical Computing, Vienna, Austria).

## 3 Results

### 3.1 Patients

As depicted in Fig. 1, 268 patients undergoing ICI treatment presented with bone metastasis. Among them, 154 patients had no record of DMAB prescription in their medical history, while 114 had received DMAB prescription at least once. Among those prescribed DMAB, 25 patients had no overlap in prescriptions, and 89 patients were prescribed both therapies concurrently. Specifically, 17 patients received ICI first followed by DMAB, while 72 patients received DMAB first followed by ICI.

The characteristics of the different patient’s populations are detailed in Table 1. There were no significant differences in age, sex, or duration of treatment observed. However, we noted a higher proportion of melanoma cases in the ICI alone group (9.7 % vs. 0 % in the ICI then DMAB group and. 1.4 % in the DMAB then ICI group), more poorly differentiated cancers in the ICI then DMAB group (13 % vs. 2.2 % in the ICI alone group and. 4.2 % in the DMAB then ICI group), and a higher proportion of Pembrolizumab treatment in the ICI then DMAB group (35 % vs. 18 % in the ICI alone group and. 8.3 % in the DMAB then ICI group). DMAB usage following ICI prescription was more frequently observed in 2018 (53 %) compared to previous years. This increase is attributed to the availability of DMAB since 2013 and the widespread adoption of ICI after 2017, particularly in the treatment of lung cancer (Table 1). Among the 154 patients receiving ICI monotherapy, 20 received zoledronic acid, including 10 having only a single infusion for the treatment of hypercalcemia. In the ICI and DMAB group (114 patients), 6 received zoledronic acid, 5 of whom had a single infusion for hypercalcemia. There was no significant difference between the two groups regarding bisphosphonate administration.

### 3.2 Combotherapy shows a trend of better survival

**Fig. 2:**
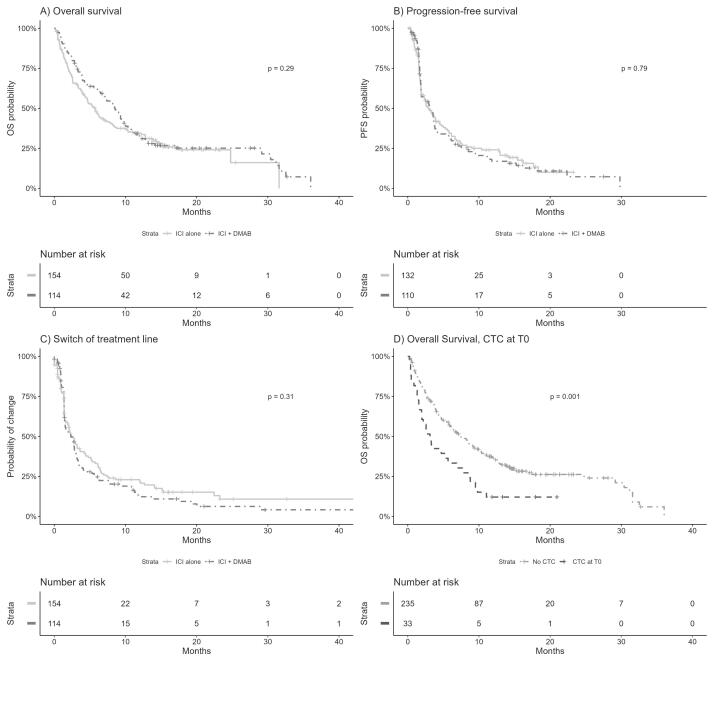
Present overall survival (OS) and progression-free survival (PFS) between ICI alone versus ICI+DMAB therapy. Kaplan–Meier analyses. (**A**) Overall survival (OS) according to DMAB prescription (ICI alone versus ICI+DMAB therapy). (**B**) Progression-Free Survival (PFS) according to DMAB prescription (ICI alone versus ICI+DMAB therapy). **(C)** Switch of treatment line according to DMAB prescription (ICI alone versus ICI+DMAB therapy). **(D)** Overall survival (OS) according to corticosteroids prescription (corticosteroids use below 10 mg/day in black versus corticosteroids use superior to 10 mg/day in grey line).

When considering the total population, there was a numerical difference in overall survival (OS) that did not reach significance. The median OS was 7.2 months in the ICI alone group and 9.1 months in the ICI+DMAB group (p = 0.29) (Fig. 2A). Similarly, there were no observed differences in terms of progression-free survival (PFS) with median PFS of 2.7 months in the ICI alone group and 2.8 months in the ICI+DMAB group (p = 0.79) (Fig. 2B). The median switching of treatment lines occurred at 2.2 months in the ICI alone group versus 2.3 months in the ICI+DMAB group (p = 0.31) (Fig. 2C). As previously reported to have a poorer prognosis outcome, we examined corticosteroids prescription (>10 mg/day of prednisone equivalent) at the initiation of ICI therapy in our population. 33 patients in the whole population had corticosteroid prescriptions before ICI initiation, and their median OS was 2.9 months compared to 9.2 months without corticosteroids (p = 0.001) (Fig. 2D). Their median PFS was 2.6 months compared to 3.8 months without corticosteroids (p = 0.15) (Fig. 2D).

**Fig. 3:**
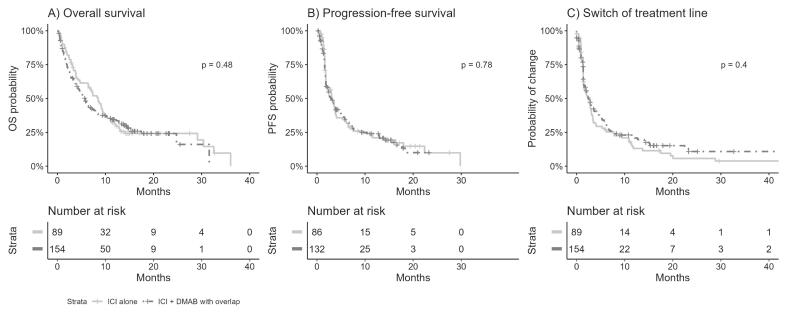
Presents os and pfs comparing the combotherapy group with ici monotherapy group. Kaplan–Meier analyses. (**A**) Overall survival (OS) according to DMAB prescription (ICI alone in black versus ICI+DMAB combotherapy in grey). (**B**) Progression-Free Survival (PFS) according to DMAB prescription (ICI alone in black versus ICI+DMAB combotherapy in grey). **(C)** Switch of treatment line according to DMAB prescription (ICI alone in black versus ICI+DMAB combotherapy in grey).

Subsequently, we focused on patients undergoing combination therapy. We found a median OS of 7.3 months in the ICI alone group and 9.2 months in the ICI+DMAB group. However, this difference did not reach significance (p = 0.48) (Fig. 3A). Similarly, the median PFS was 2.7 months in the ICI alone group and 3.1 months in the ICI+DMAB group, which did not reach significance (p = 0.78) (Fig. 3B). Finally, the median switching of treatment lines occurred at 2.2 months in the ICI alone group versus 2.3 months in the ICI+DMAB group (p = 0.4) (Fig. 3C).

### 3.3 ICI then DMAB sequence as a better outcome

**Fig. 4:**
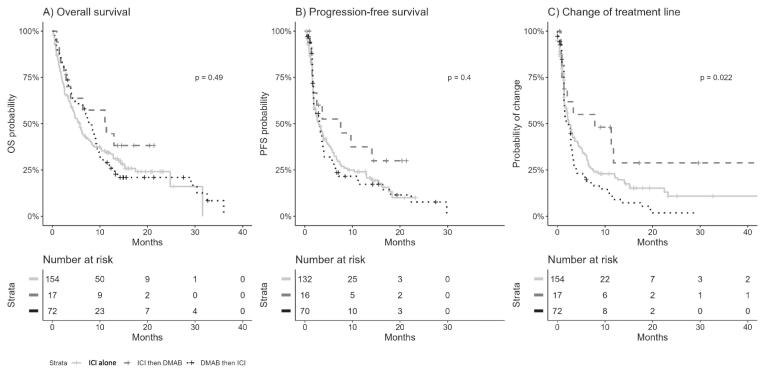
Present os, pfs, and treatment line change between the sequence group. Kaplan–Meier analyses. (**A**) Overall survival (OS) according to DMAB prescription (ICI alone in grey line versus ICI then DMAB in black intermittent line versus ICI then DMAB in black dotted line prescription). (**B**) Progression-Free Survival (PFS) according to DMAB prescription (ICI alone in grey line versus ICI then DMAB in black intermittent line versus ICI then DMAB in black dotted line prescription). **(C)** Switch of treatment line according to DMAB prescription (ICI alone in grey line versus ICI then DMAB in black intermittent line versus ICI then DMAB in black dotted line prescription).

Furthermore, we examined the sequence of DMAB prescription compared to ICI initiation. The median OS of the ICI alone group was 7.2 months, compared to 9.1 months in the DMAB then ICI group and 10.9 months in the ICI then DMAB group. However, this trend was not significant (p = 0.49) (Fig. 4A). Similarly, the median PFS was 2.7 months in the ICI alone group, 2.7 months in the DMAB then ICI group, and 7.2 months in the ICI then DMAB group. These differences were not significant (p = 0.4) (Fig. 4B). The median switching of treatment lines was 2.2 months in the ICI alone group, 2.2 months in the DMAB then ICI group, and 8.2 months in the ICI then DMAB group, reaching significance (p = 0.022) (Fig. 4C).

### 3.4 Osteonecrosis of the jaws

In our population, we identified 10 ONJs (3.7 %). Two ONJ patients received ICI only. All the others received antiresorptive agents and/or antiangiogenics. There was a higher proportion of ONJ in patients treated with DMAB (7.0 % vs. 1.3 % in the ICI group alone). One ONJ patient received only DMAB and 7 received both DMAB and antiangiogenic therapy. Only 1 ONJ patient received bisphosphonate but he was previously treated by antiangiogenic therapy and after by denosumab (21 SC infusions before developing ONJ). There was a higher prevalence of ONJ in patients treated with antiangiogenic agents (8.4 % vs. 1.6 %).

## 4 Discussion

In this study, we observed a better outcome when using ICI before DMAB initiation compared to the absence of DMAB use and DMAB before ICI initiation in terms of switching treatment line in a bone metastatic cohort of patients treated with ICI (p = 0.022). We also observed trends without significance for OS and PFS favoring ICI use before DMAB initiation but not reaching significance.

As our results are negative for OS and PFS in our bone metastatic cohort of patients, it differs from several clinical case reports that were encouraging in this situation [13], [15], [23] and that supported an unconditional synergy between DMAB and ICI. Retrospective series were also quite encouraging. Afzal et al. reported in 37 patients treated with ICI for malignant melanoma a median OS of 22.8 (16–36) months (26 patients treated with only ICI) compared to 57.0 (7.6-NA) months (11 patients with ICI and DMAB) [19]. This was not significant because of a small sample size in their cohort. Angela et al. also looked at bone metastatic malignant melanoma and found an ORR of 53.9 % for triple therapy (anti-PD1, anti-CTLA4, and DMAB) versus 43.8 % for double therapy (anti-PD1 and anti-CTLA4) showing once again a trend for the superiority of DMAB use in this context [20]. Finally, Liede et al. found in a cohort of non-small cell lung cancer (NSCLC) and malignant melanoma a better OS in patients treated with a longer duration of DMAB treatment in NSCLC patients (19.9 weeks for patients treated > 14 weeks with ICI versus 8.3 weeks for patients treated < 6 weeks) [23]. Asano et al. also recently published in a bone metastatic NSCLC cohort of 55 patients a positive effect on prognosis of bone targeting agent use in combination with ICI [24].

On the other hand, our work was also built to answer the question of the interest of a sequence of use between DMAB and ICI. Indeed, pre-clinical studies also described an advantage of using ICI before DMAB use in animal models [12]. As previously published, Ahern et al. found a dramatic effect of anti-PD1 and anti-CTLA4 use before DMAB on tumor growth whereas the first use of DMAB then anti-PD1 and anti-CTLA4 were similar to placebo [12]. As they propose, ICI can cause upregulation of RANKL expression in T cells which could promote interaction with immunosuppressive RANK-expressing cells in the tumor microenvironment. Denosumab might block this process and thereby relieve immunosuppression. RANK is also expressed in dendritic cells that can mediate immunosuppression by blocking T cell activation [9], [10]. The use of DMAB might relieve this suppression leading to an increased number of active T cells, which result in an enhanced immune response [9], [10]. Finally, DMAB could have a direct effect on RANKL-expressing immune cells via cell depletion or reverse signaling [2]. As observed in these papers, the sequence of use seems to be more relevant than looking only at combination therapy. In our work, the only situation that shows a clear improvement at least numerically is the use of ICI before DMAB initiation.

In our population, we also observed the prescription of zoledronic acid. Notably, there was no significant difference between the two groups regarding zoledronic acid use in the ICI monotherapy and ICI-DMAB populations. Given that only 26 patients received zoledronic acid, with 15 of them having a single infusion, we are unable to determine the impact of zoledronic acid use in our population, as the analysis is underpowered. Moreover, monthly zoledronic acid infusions are recommended in such cases [25]. Previous phase III trials evaluating the bone-health benefits of bisphosphonates did not demonstrate improved overall survival (OS) in patients [26], [27], [28]. However, post hoc analyses have suggested that zoledronic acid treatment was associated with improved survival in lung cancer patients, particularly those with elevated bone resorption markers [29]. Mechanistically, zoledronic acid may exert effects not only as an anti-resorptive agent on vicious circle but also by influencing immune cell subsets, such as Vγ9Vδ2 T cells [30]. Recent research by Frieling et al. demonstrated that these cells induce regression of intratibial human C4-2B prostate tumors in immunodeficient mice, with the amino-bisphosphonate zoledronate further enhancing the rate of tumor regression in these models [31].

We also describe a poorer prognosis of corticosteroid use (>10 mg/day of prednisone equivalent) before ICI initiation. This is, to our knowledge, the first description of this phenomenon in a bone metastatic cohort that underwent ICI therapy. One in vivo study showed that PD-1 blockade enhanced neoantigen-specific CD8 + T cell responses leading to tumor regression [32]. With concurrent immunotherapy and steroid use, there was a reduction in low-affinity memory CD8 + T cells and blunted antitumor responses [33]. A retrospective study of > 2,000 patients on immunotherapy for advanced melanoma, NSCLC, and urothelial cancer is the most important register to focus on this peculiar subject [34]. Baseline systematic steroid use (defined as ≤ 14 days prior to, and up to 30 days after the start of immunotherapy) was associated with a 23 %−47 % increased risk of death compared with no use. Patients on baseline steroids were more likely to have advanced staging at diagnosis, distant metastases (including brain and liver), and poorer Eastern Cooperative Oncology Group (ECOG) performance scores. However, baseline steroids remained a significant factor even in multivariable analysis, suggesting a causal link. In our cohort of bone metastatic patients, results are in line with this conclusion, with an observed shorter median OS (2.9 months versus 9.2 months) despite we did not describe the reason for steroid use in our cohort.

ONJ is defined by the presence of exposed mandibular or maxillary jawbone for at least 8 weeks [35]. This disease is observed under BP therapy but in some case ONJ occur in BP-naive patients, notably those who received DMAB or antiangiogenic therapies, which are now well-known risk factors [36], [37], [38], [39], [40], [41]. We currently have accumulating data on ONJ incidence in populations treated with antiresorptive agents, whether osteoporotic (0 to 90 per 100,000 patient-years) [42], [43], [44], [45] or oncological (0 to 12,222 per 100,000 patient-years) [46], [47], [48], [49]. Several cases of ONJ associated with the use of ICI without antiresorptive, radiotherapy or antiangiogenic therapies have been reported [50].

The main limitation of our study is its retrospective nature and the lack of patients (n = 17) in the group ICI then DMAB prescription that gave trends but not significance for OS and PFS favoring ICI then DMAB sequence compared to ICI alone and DMAB then ICI use. Moreover, our population includes patients who were treated with ICI between 4 to 8 years ago and many oncology management protocols evolved.

The use of ICI seems to be taking place earlier in the management process. In our population, many patients received ICI after several lines of treatment, with an unfavorable prognosis and reduced life expectancy, making it difficult to identify any difference in overall survival.

It therefore seems important to perform new studies with stronger methodology, such as prospective studies. Some of these are already underway (DENIVOS [51], POPCORN [52]. It also seems interesting to study the association between ICI and DMAB with current practices, where the place of ICI use is different from a few years ago, and better define the interest of the association of bone-targeted agents such as denosumab with ICI more than a delayer of bone skeletal-related event preventer.

## CRediT authorship contribution statement

**E. Mabrut:** Writing – original draft, Formal analysis, Data curation. **S. Mainbourg:** Methodology, Formal analysis, Data curation. **J. Peron:** Methodology, Formal analysis, Conceptualization. **D. Maillet:** Writing – review & editing, Data curation. **S. Dalle:** Writing – review & editing, Data curation. **C. Fontaine Delaruelle:** Writing – review & editing, Data curation. **E. Grolleau:** Writing – review & editing, Data curation. **P. Clezardin:** Writing – review & editing. **E. Bonnelye:** Writing – review & editing, Formal analysis. **C.B. Confavreux:** Writing – review & editing, Validation, Investigation, Formal analysis, Conceptualization. **E. Massy:** Writing – review & editing, Validation, Investigation, Formal analysis, Conceptualization.

## Declaration of competing interest

The authors declare that they have no known competing financial interest or personal relationship that could have appeared to influence the work reported in this paper.
